# Vascular endothelial growth factor expression correlates with serum CA125 and represents a useful tool in prediction of refractoriness to platinum-based chemotherapy and ascites formation in epithelial ovarian cancer

**DOI:** 10.18632/oncotarget.4427

**Published:** 2015-06-10

**Authors:** Samar Masoumi-Moghaddam, Afshin Amini, Ai-Qun Wei, Gregory Robertson, David L. Morris

**Affiliations:** ^1^ Department of Surgery, St. George Hospital, The University of New South Wales, Kogarah, Sydney, NSW, Australia; ^2^ Department of Orthopedic Surgery, St. George Hospital, The University of New South Wales, Kogarah, Sydney, NSW, Australia; ^3^ Department of Gynecology Oncology, St. George Hospital, The University of New South Wales, Kogarah, Sydney, NSW, Australia

**Keywords:** ascites, CA125, epithelial ovarian cancer, fibroblast growth factor, chemorefractoriness

## Abstract

There is an increasing need for the identification of novel biological markers and potential therapeutic targets in epithelial ovarian cancer (EOC). Given the critical role of growth factors in the biology of EOC, we aimed in the present study to evaluate the intratumoral expressions of vascular endothelial growth factor (VEGF) and fibroblast growth factor (FGF) proteins and their clinical relevance in a cohort of 100 patients with EOC. All patients received platinum-based chemotherapy after surgery. A comparative immunohistochemical study of normal ovarian and EOC tissues showed that both growth factors were expressed at higher levels in tumor samples. In our statistical analysis, while no association existed between the FGF expression status and the clinicopathological characteristics of patients, intratumoral VEGF was identified as a potential biomarker for the prediction of ascites formation. In addition, the expression status of VEGF appeared to independently predict overall survival and response to chemotherapy. Furthermore, a direct association was demonstrated between the pre-treatment VEGF expression and serum CA125 after three cycles of chemotherapy. In sum, we report for the first time to our knowledge the correlation between intratumoral VEGF and serum CA125 in EOC. Our data also shows the prognostic value of VEGF expression in EOC. These results suggest the potential value of intratumoral VEGF in patient stratification. Dual inhibition of VEGF and CA125 might bring about a better outcome for patients with EOC.

## INTRODUCTION

Epithelial ovarian cancer (EOC) is the deadliest gynecologic cancer in the United States. [1] Most women with EOC have advanced disease at diagnosis. The late presentation and widespread abdominal metastasis account for the high death rate. Despite invasive surgery and platinum-based cytotoxic chemotherapy as the standard of care for advanced disease, episodes of recurrent disease, progressively shorter disease-free intervals and resistance to chemotherapy will develop in most cases. [2] Hence, there is an increasing need for the development of novel biomarkers for diagnostic, therapeutic and prognostic purposes in EOC. In addition, discovery of reliable stratification biomarkers for identification of patients who may benefit from a given therapy is of great importance. [3]

As one of the most studied growth factors, vascular endothelial growth factor (VEGF) contributes to the growth and progression of EOC and associated ascites formation. [4] Overexpression of intratumoral VEGF has been reported to correlate with poorer prognosis [5–7] and suggested as an independent predictor of patient survival. [8] Similarly, fibroblast growth factor (FGF) has been shown to stimulate proliferation, migration, invasion and angiogenesis of ovarian cancer cells. [9–12] An increase in the expression of FGF has also been reported in EOC. [13, 14] The clinical relevance of the FGF expression, however, has been controversial. In the present study, we evaluated the intratumoral expression of these growth factors in patients with EOC, investigated their relevance with clinicopathological characteristics, and explored their correlation with serum CA125 after adjuvant platinum-based chemotherapy. Here, we report that VEGF expression status of our cohort could predict refractoriness to chemotherapy, overall survival and ascites formation, and was directly associated with serum CA125.

## RESULTS

### Patients’ data

The demographic and clinicopathological characteristics of the participants are summarized in Table 1. Except one, all patients had high pre-operative serum CA125 levels (> 35 U/ml).

**Table 1:** Demographic and clinicopathological characteristics of the cohort

Characteristic		Categorization	Patients (n = 100, %)
Age (year)	Range: 35-84	≤ 50	16
	Median: 62	> 50	84
Menopause		Yes	92
		No	8
Histological subtype		High-grade serous	63
		Low-grade serous	18
		High-grade endometrioid	2
		Low-grade endometrioid	2
		Mucinous	2
		Clear cell	5
		Others	8
FIGO stage		I-II	14
		III-IV	86
Extent of residual tumour		None	48
		<1 cm	35
		1-2 cm	0
		>2 cm	17
Serum CA125 after three cycles		Normal	53
		High	47
Clinical response to platinum chemotherapy		Sensitive	79
		Resistant	21
post-treatment ascites		Yes	42
		No	58

### VEGF and FGF proteins are upregulated in EOC

**Figure 1:**
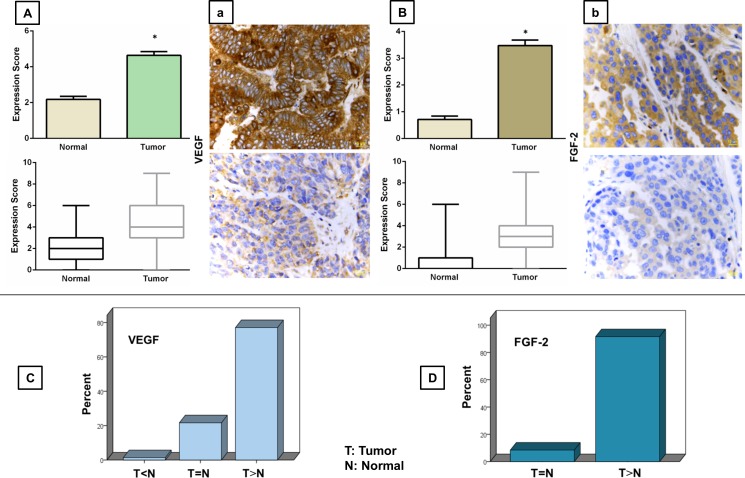
Immunohistochemical analysis of VEGF (A-a) and FGF (B-b) expressions in human epithelial ovarian cancer tissue Upper graphs represent upregulation of VEGF **A.** and FGF **B.** proteins in EOC as compared to the normal ovarian tissue. Data are represented as mean expression score ± SE (top row) and maximum and minimum expression score (bottom row). Significant values (< 0.05) are marked by asterisks. Micrographs (a-b) show high (top row) and low (bottom row) levels of the immunohistochemical expression of the proteins of interest in the EOC tissue (magnification = 40x). Lower graphs demonstrate the percentage of cases with higher (T > N), lower (T < N) or equal (T = N) expression of VEGF **C.** and FGF **D.** as compared to their matched normal ovarian tissue.

Immunohistochemical reactivity of VEGF and FGF in the epithelial cells was mainly confined to the cytoplasm. A comparison of the expression levels of these markers in tumor and normal tissue revealed a significant upregulation of VEGF (*p* < 0.0001) and FGF (*p* < 0.0001) in EOC (Figure 1A–1B). Mean expression scores of VEGF and FGF in tumor samples were 4.64±0.21 (range; 0-9) and 3.47±0.20 (range; 0-9), respectively, as compared to 2.18±0.17 (range; 0-6) and 0.71±0.12 (range; 0-6) in normal ovarian tissues.

Using the predefined binary cut-off points, tumor samples were then classified into high- and low-expressing groups. As a result, 59 out of 100 cases were identified as patients with high expression of VEGF (score > 3.5). With regard to FGF, 31 out of 95 cases were found to be high-expressing (score > 3.5). Due to the variability of the protein expression in different samples, we next compared the staining scores of VEGF and FGF in cancer and matched normal tissues from the same patient for a more meaningful assessment. Our analysis indicated higher expression levels of VEGF and FGF in 77 and 91.5 percent of tumor tissues, respectively, as compared with their matched normal tissues (Figure 1C–1D).

To explore a possible correlation between the expression of the growth factors and tumor type and subtype, we subsequently performed a subgroup analysis. The type and subtype distribution of our cohort is listed in Table 1. Our data indicated the mean VEGF expression scores of 4.62±0.24, 4.72±0.56, 3.00±0.01, 4.50±1.50, 2.00±2.00, 4.60±1.12 and 5.75±0.84 for high-grade serous, low-grade serous, high-grade endometrioid, low-grade endometrioid, mucinous, clear cell, and unclassified groups, respectively. Comparing the VEGF expression values among different subtypes, we observed no significant differences between high- and low-grade serous cancers (p:0.85), as well as between high- and low-grade endometrioid cancers (p:0.42). We then compared the mean VEGF expression scores of high-grade serous tumors with those of the other types/subtypes. Although the mean expression scores of VEGF in mucinous, high-grade endometrioid and low-grade endometrioid tumors was lower than those in high-grade serous tumors, the difference did not reach the statistical significance level (mucinous, p: 0.069; high-grade endometrioid, p:0.249; low-grade endometrioid, 0.933). As regards the clear cell and others, no statistically meaningful differences were observed (p:0.984 and 0.137, respectively). Likewise, a similar subgroup analysis of FGF expression yielded no statistically significant difference among the individual types and subtypes.

### VEGF, but not FGF, correlates with serum CA125 and predicts clinical response to platinum-based chemotherapy of EOC

To investigate the clinical relevance of the expression of our proteins of interest, we initially explored the possible correlations of intratumoral VEGF and FGF with serum CA125 levels after three cycles of chemotherapy, as well as with the development of refractoriness to carbotaxol treatment. Our data analysis revealed a direct correlation between the expression of VEGF in tumor tissue and both serum CA125 (p:0.032, correlation coefficient = 0.215) and chemorefractoriness (*p* = 0.005, correlation coefficient = 0.280). The mean expression score of VEGF in patients who were sensitive to platinum chemotherapy was 4.47±0.23 as compared with 5.29±0.45 in those with refractory disease. As regards FGF, however, the mean expression scores of the protein in both groups were similar (3.51±0.24 and 3.35±0.35 in platinum-sensitive and non-sensitive groups, respectively). Intratumoral FGF did not show a significant association with serum CA125 (p:0.877) or chemorefractory disease (p:0.780), either.

Using univariate and multivariate binary logistic regression analyses, we next evaluated the significance of VEGF and FGF expressions in predicting response to chemotherapy with carboplatin and taxol. While univariate analysis yielded no statistically significant predictive value for FGF (p:0.778), VEGF was identified as a predictor of response to chemotherapy (HR = 0.18; 95% CI, 0.04-0.65; p:0.010). Other parameters with significant predictive value in univariate analysis included tumor subtype (HR = 0.18; 95% CI, 0.04-0.88; p:0.034) and residual disease (HR = 0.22; 95% CI, 0.06-0.76; p:0.016). Tumor VEGF, subtype and residual disease retained their independent significance in multivariate analysis, too (VEGF: HR = 0.19; 95% CI, 0.04-0.77; p:0.021; tumor subtype: HR = 0.10; 95% CI, 0.01-0.60; p:0.012; residual tumor: HR = 0.13; 95% CI, 0.03-0.56; p:0.007).

### VEGF, but not FGF, predicts development of post-treatment ascites in EOC

In our cohort, 65% of cases had proven ascites either at the time of diagnosis or after treatment. In these patients, the mean expression scores of VEGF and FGF were 5±0.24 and 3.52±0.26, respectively. When these expression levels were compared with the corresponding values in non-ascites group (mean expression scores of 3.97±0.36 and 3.38±0.34 for VEGF and FGF, respectively), a significant increase in intratumoral VEGF was revealed (*p* values of 0.019 and 0.743 for VEGF and FGF, respectively).

In the subgroup with ascites at the time of diagnosis, the mean VEGF and FGF expression scores were 4.83±0.26 and 3.58±0.30, respectively, as compared with 4.41±0.34 and 3.36±0.27 in non-ascites group. A similar comparison between patients who did and did not develop ascites after treatment indicated significantly higher VEGF expression levels in the former (5.17±0.32 vs 4.26±0.27; p:0.033). In this subgroup, however, the increase in the FGF expression was not statistically significant (3.61±0.34 vs 3.39±0.26; p:0.606). Evaluating the association between the expression of the two growth factors and history of ascites, we demonstrated a direct significant correlation between the VEGF expression and ascites formation (ascites at the time of diagnosis; p:0.036, correlation coefficient = 0.210, and post-treatment ascites; p:0.003, correlation coefficient = 0.297). With respect to FGF, no significant association with ascites formation was found.

The predictive value of VEGF and FGF expressions with regard to the development of post-treatment ascites was further evaluated employing logistic regression analysis. Univariate test revealed the significance of the VEGF expression for predicting the post-treatment development of ascites (HR = 0.27; 95% CI, 0.11-0.65; p 0.004). Other clinicopathological variables with predictive value included stage (HR = 0.08; 95% CI, 0.01-0.67; p:0.020), ascites at diagnosis (HR = 0.23; 95% CI, 0.09-0.55; p:0.001) and refractory disease (HR = 0.15; 95% CI, 0.05-0.46; p:0.001). In multivariate analysis, ascites at diagnosis (HR = 0.22; 95% CI, 0.07-0.65; p:0.006) and refractory disease (HR = 0.12; 95% CI, 0.03-0.48; p:0.003) were identified as independent predictors of post-treatment ascites. However, the independent predictive value of VEGF and stage was not statistically significant (*p* values of 0.174 and 0.066, respectively).

### VEGF predicts overall survival in EOC

**Figure 2:**
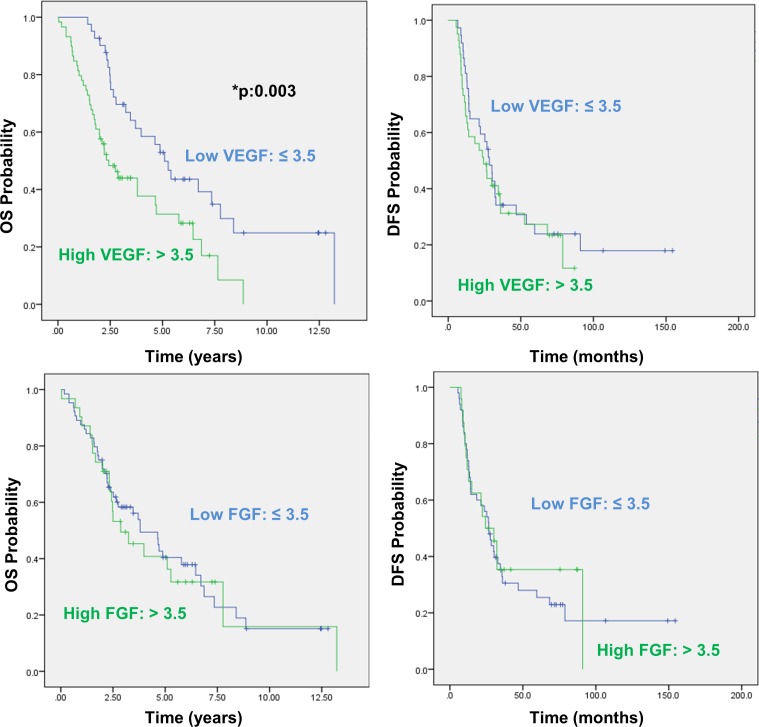
Overall and disease free survival analysis with regard to VEGF and FGF expression in EOC Top and bottom graphs demonstrate Kaplan-Meier curves for overall survival (OS) (left) and disease free survival (DFS) (right) probabilities in correlation with VEGF and FGF expressions, respectively. Significant values (< 0.05) are marked by asterisks.

Next, we used Kaplan-Meier method to investigate the relationship between the expression of the growth factors in tumor tissue and patient outcome, including overall survival (OS) and disease free survival (DFS). OS was recorded as months of survival after surgery, or months from surgery to death. The median OS was 2.43 years in patients with a high VEGF score as compared with 5.095 years in patients with a low VEGF score, indicating a significant decrease in median OS of high-expressing group (p:0.003) (Figure 2). Table 2 shows 3-, 5- and 8-year OS rates with regard to the VEGF expression. As regards FGF, the difference between the median OS for high-expressing and low-expressing groups (2.86 and 3.80 years, respectively) was not statistically significant (p:0.78). In relation to DFS, a similar analysis did not yield significant results for either VEGF (p:0.511) or FGF (p:0.723).

**Table 2:** Overall survival rate with regard to VEGF expression

Overall Survival	Low VEGF	High VEGF
3-year	70%	44%
5-year	53%	32%
8-year	30%	8%

The prognostic value of tumor VEGF and FGF was then assessed along with the clinicopathological characteristics of the participants in univariate and multivariate analyses. In univariate analysis, low VEGF appeared to be a significant predictor of increased OS (HR = 0.46; 95% CI, 0.27-0.77; p:0.003), but failed to show predictive value for DFS (p:0.512). Low stage (HR = 0.28; 95% CI, 0.11-0.71; p:0.008), absence of ascites at the time of diagnosis (HR = 0.59; 95% CI, 0.36-0.98; p:0.045) and low residual disease after cytoreductive surgery (HR = 0.44; 95% CI, 0.23-0.84; p:0.013) were also identified as parameters which significantly predicted increased OS. With respect to FGF, however, the protein expression did not hold prognostic value in univariate analysis (*p* values of 0.783 and 0.723 for OS and DFS, respectively). Factors with predictive significance in univariate analysis were then subjected to multivariate Cox's proportional hazards analysis. As a result, low VEGF retained its significance for OS (HR = 0.46; 95% CI, 0.27-0.79; p:0.005) and appeared to be an independent prognostic biomarker along with stage (HR = 0.27; 95% CI, 0.10-0.72; p:0.009) and residual disease (HR = 0.36; 95% CI, 0.19-0.71; p:0.003).

### CA125 correlates with ascites development and predicts overall survival, development of ascites and refractoriness to carbotaxol in EOC

Through performing Spearman's test, we then found a direct significant correlation between serum levels of CA125 after the third course of chemotherapy and the development of post-treatment ascites (p:0.003, correlation coefficient = 0.295). Our univariate and multivariate analyses into the prediction of post-treatment ascites formation revealed CA125 as a dependent predictor (univariate, HR = 0.29; 95% CI, 0.12-0.67; p:0.004; multivariate, p:0.349), and identified ascites at diagnosis (HR = 0.21; 95% CI, 0.07-0.60; p:0.004) and refractory disease (HR = 0.13; 95% CI, 0.03-0.55; p:0.006) as independent predictive factors. Kaplan-Meier test estimated a median OS of 6.46 years in patients whose CA125 levels were back to normal just after the third cycle of chemotherapy versus 2.31 years in those with abnormal CA125 (*p* < 0.0001). The predictive value of CA125 for OS was further analyzed by Cox's tests (univariate, HR = 0.34; 95% CI, 0.21-0.57; *p* < 0.0001; multivariate, HR = 0.36; 95% CI, 0.22-0.61; *p* < 0.0001), wherein CA125, stage (HR = 0.36; 95% CI, 0.14-0.95; p:0.039) and residual disease (HR = 0.40; 95% CI, 0.20-0.77; p:0.006) were identified as independent predictors of OS. Serum CA125 also showed dependent predictive value for DFS (univariate, HR = 0.52; 95% CI, 0.30-0.89; p:0.017, multivariate, p:0.077). Finally, it was found to be an independent predictor of chemorefractory disease, too (univariate, HR = 0.05; 95% CI, 0.01-0.26; *p* < 0.0001; multivariate, HR = 0.01; 95% CI, 0.002-0.14; *p* < 0.0001). In this regard, tumor subtype and residual disease represented the other independent predictors (*p* values of 0.006 and 0.003, respectively).

## DISCUSSION

The significance of VEGF in EOC is well established. While playing an important role in the physiology of normal ovaries, VEGF has a major contribution to the growth and development of EOC mainly through the induction of tumor angiogenesis and enhancement of vascular permeability. [4] Moreover, it has been argued that VEGF might directly promote the growth and proliferation of EOC cells through an autocrine loop. [15–17] Preclinical studies have shown that the overexpression of VEGF can transform normally functional ovarian epithelium into neoplastic, ascites-producing tissue. [18, 19] In agreement, we observed in the present study that the VEGF expression was upregulated in EOC, and independently predicted OS and response to chemotherapy with carbotaxol. Since the data on the prognostic implication of circulating VEGF in blood samples is more heterogeneous and needs further investigation and validations, [20–22] the detection of intratumoral VEGF was aimed in the present study. Our results are in line with the earlier immunohistochemical studies reporting on the elevated expression of VEGF in EOC. [8, 23–28] Also in agreement are the clinical reports similarly suggesting that the intratumoral expression of VEGF directly correlates with disease progression [29] and poor survival [23, 25, 27, 30, 31] and serves as an independent prognostic factor [8] or a biomarker of response to platinum-based chemotherapy [26]. Likewise, serum VEGF has been proposed as a diagnostic biomarker and a predictor of prognosis in patients with EOC [32–34]. Given its known contribution to the pathophysiology of solid tumors, VEGF has been the focus of targeted therapies. In EOC, however, prolongation of survival and cure with VEGF-targeted agents remains elusive. In this regard, the addition of bevacizumab to standard chemotherapy evaluated in four randomized, double-blind, phase III trials, both as front-line treatment (GOG-0218 and ICON7) and in patients with recurrent disease (OCEANS and AURELIA), has shown marginal benefits with regard to DFS and essentially no statistically significant improvements in OS. [35] Moreover, preclinical data [36, 37] has suggested that VEGF blockade in certain circumstances may confer a more aggressive tumor phenotype. [4, 38]

In addition, the role of VEGF in peritoneal dissemination of EOC and malignant ascites formation is well documented. [4] VEGF levels were reported to be markedly elevated in EOC-associated malignant ascites compared with nonmalignant ascitic fluids, [39] and of prognostic significance. [40] Blockade of VEGF has also been shown to disrupt ascites formation. [41, 42] In agreement, we observed a direct association between the intratumoral VEGF expression and the development of post-treatment ascites which was further shown to hold predictive value for post-treatment formation of ascites.

FGF is also among growth factors implicated in the pathophysiology of EOC. FGF has been reported to stimulate proliferation, migration and invasion of EOC cells *in vitro* and to promote angiogenesis *in vivo* [9–12]. Increased expression of FGF (mRNA and/or protein) in tumor tissue, [13, 14] raised concentration of FGF in serum and/or ascetic fluid, [43] or elevated levels of FGF in both tumor and serum [44] have been reported in EOC. In the present study, we observed that FGF is upregulated in EOC tissue. The increased intratumoral FGF, however, was not associated with the clinicopathological features or clinical outcome. The clinical relevance of FGF in EOC is still controversial. Gan et al. reported that high FGF expression was inversely correlated with sensitivity to paclitaxel and was a strong predictor of resistance to the drug. [45] In contrast, Obermair et al. [46] and Secord et al. [47] have reported inverse correlation of intratumoral FGF with tumor progression and poor survival.

Our data also uncovered a correlation between intratumoral VEGF and serum CA125 levels after three cycles of chemotherapy. CA125 is a high-molecular-weight glycoprotein which is extensively expressed by EOC tumors. [48, 49] Candido Dos Reis et al. reported that serum CA125 and cystic VEGF served well to differentiate benign ovarian tumors from EOC. In line with our results, they also found a significant correlation between serum levels of CA125 and matched cyst levels of VEGF. [50] They assumed this finding to be an evidence for a new hypothesis according to which VEGF-induced angiogenesis and enhanced vascular permeability in patients with EOC result in the release of CA125 into the circulation. Rosen et al. found in the CA125-deficient subgroup of EOC patients that intratumoral VEGF was expressed in a large fraction in the absence of CA125. [49] These observations, along with our findings, can justify the feasibility of tumor VEGF as a potential tumor marker in EOC irrespective of CA125 levels and provides evidence for the potential correlation between the two markers.

Similar to VEGF, CA125 was found in the present study to predict and correlate with ascites development, and also served as an independent predictor of OS and chemorefractoriness. CA125 plays an important role in the biology of EOC by facilitating peritoneal metastasis of tumor cells and enhancing their aggressive behavior. [51, 52] It is upregulated in mesothelial epithelium during ascites growth of EOC, which is itself under control of growth factor signaling. [53] Du et al. observed a markedly higher rate of lymph node metastasis and tumor volume as well as upregulation of CA125 in the involved lymph nodes by VEGF-overexpressing EOC xenografts as compared to control. [54] In agreement with earlier reports, our results thus further highlight the clinicopathological relevance of tumor VEGF and serum CA125 and, more importantly, reveal a correlation between the two in EOC. On this basis, the development of a treatment modality with dual suppressing activity on VEGF and CA125 might be of potential value in novel approaches to EOC.

In sum, we report the upregulation of VEGF and FGF in EOC. Our data also supports the VEGF involvement in or association with the development of EOC-induced malignant ascites. In our study, VEGF and CA125 appeared to predict OS and response to adjuvant platinum-based chemotherapy. Treatment strategies with potential ability to inhibit the expression of VEGF and CA125 might bring about a better outcome for patients with EOC.

## PATIENTS AND METHODS

### Clinical cases and surgical specimens

A review of the clinical records of patients with EOC from two specialized centers (St. George Hospital; St George Private Hospital, Sydney, Australia) between January 2001 and December 2012 was performed. Institutional review board approval for this analysis was obtained. A total of 100 patients with a histological diagnosis of primary EOC who had been treated with standard surgical procedure (staging laparotomy/cytoreductive surgery) plus adjuvant systemic chemotherapy (paclitaxel + carboplatin as formulated below) and had a complete follow-up history till June 2014 (end of the study) were included in this study and their informed consents were obtained.

Adjuvant chemotherapy regimen

Paclitaxel (175 mg/m^2^, iv over 3 hours) + carboplatin (total dose calculated by Calvert formula*, iv over 15-60 minutes) × 6 cycles

* Total carboplatin dose (mg) = Target area under concentration vs time curve (AUC) × (GFR +25)

Archived formalin-fixed, paraffin-embedded material from surgically resected primary EOC specimens containing tumor and the matched normal tissue (from the contralateral ovary or normal portion of the affected one) was employed. A confirmatory review of pathology was performed. Ovarian neoplasms were histologically classified according to the World Health Organization (WHO) classification system. [55] Serum CA125 level of more than 35 U/ml after the third chemotherapy cycle was considered high. [56, 57] Patients whose CA125 level fell within the reference range were classified as cases with normal CA125 after three cycles. [58] The final staging of the disease was determined on the basis of a combination of surgical and pathological findings in accord with the old Federation of Gynecology and Obstetrics (FIGO) guidelines. [59] In this study, in line with general categorization adopted for EOC, “platinum sensitivity” refers to disease recurrence 6 months or more after cessation of the prior platinum-containing chemotherapy, and “platinum resistance” refers to a response to platinum-based chemotherapy followed by relapse less than 6 months after chemotherapy is stopped. “Platinum-refractory disease” refers to a lack of response or to progression while on platinum-based chemotherapy. For patients with platinum-sensitive disease, retreatment with a platinum or platinum-containing combination, such as carboplatin, was considered. For cases demonstrating platinum-refractory or platinum-resistant disease who did not consent for clinical trials, the goals of treatment was to improve quality of life by extending the symptom-free interval, by reducing symptom intensity, and by increasing progression-free interval, and, if possible, to prolong life. Single-agent paclitaxel, liposomal doxorubicin or tamoxifen were considered as reasonable treatment options. In our cohort, the regimens for patients with resistant or recurrent disease did not include any antiangiogenic agents such as bevacizumab. Secondary cytoreduction was performed in case of obstruction or in any other conditions when the multidisciplinary committee advocated the surgery. For FGF, the existing difference in the total number of patients resulted from the inadequacy of cancer tissue remaining in the archival blocks at the time of the study. Patients who developed ascites any time after the completion of 6 cycles of adjuvant treatment were classified as cases with ascites after adjuvant treatment.

### Immunohistochemical staining

Five-micrometer sections were prepared from the paraffin blocks and immunostaining was performed as described previously [60]. Briefly, the sections were deparaffinized and microwaved in 10 mM sodium citrate buffer (DAKO A/S, Glostrup, Denmark) at pH 6.0 for 10 min at 750 W for antigen retrieval. Thereafter, the samples were incubated with 3% hydrogen peroxide (Sigma-Aldrich, St. Louis, MO, USA) and DAKO blocking buffer (EnVision Plus Kit, DAKO A/S, Glostrup, Denmark), respectively. This was followed by overnight incubation at 4° C with primary antibodies (Santa Cruz Biotechnology Inc., Santa Cruz, CA, USA). Binding of the primary antibody was detected by incubating the samples with appropriate secondary antibody (EnVision Plus Kit, DAKO A/S, Glostrup, Denmark) for 30 min and then with diaminobenzidine chromogen for 5 min. The sections were then counterstained with hematoxylin (Sigma-Aldrich, St. Louis, MO, USA). Prostate and breast cancer tissues were included as positive controls for VEGF. Tonsil and testis tissues were used as positive controls for FGF. As regards the negative controls, the same tissues as the positive controls were used but the primary antibodies were replaced with the primary antibody diluents.

### Evaluation of expression reactivity

To evaluate the staining of the epithelial cells, semi-quantitative scoring was performed according to the method used by Mattern et al. and Terris et al. [61, 62] This scoring method enables the determination of both the intensity of the immunosignal and the percentage of cells showing positive staining. The percentage of positive cells was scored as: no positive cells (0); 1-25% (1); 26-50% (2); and 50% > (3). The intensity of the staining was scored as: no staining (0); weak (1); moderate (2); strong (3). Using the following formula, the immunohistochemical scores, ranged between 0 and 9, were calculated by at least two observers blinded to patient outcome: immunohistochemical score = staining positivity score × staining intensity score.

### Statistical analysis

All statistical analyses were conducted using the statistical package SPSS, version 22 (SPSS Inc., USA). Student *t*-test was used for comparing the actual difference between two means. Spearman's correlation coefficient test was performed to evaluate the associations between the clinicopathological parameters and the expressions of the studied growth factors. The binary cut-off points of the markers were identified using the Classification and Regression Tree (CART) algorithm. This resulted in the classification of the immunohistochemically scored tissues into low (score ≤3.5) and high (score > 3.5) groups. The study end points of disease-free survival (DFS) and overall survival (OS) were analyzed using the Kaplan-Meier method. Univariate and multivariate logistic regression analyses were conducted to ascertain the effect of the studied growth factors and other clinicopathological variables on the likelihood of the development of post-treatment ascites and chemorefractory disease. A *p* value of < 0.05 was considered statistically significant for all analyses.
